# Host Country Views of Short-Term Medical Missions: Community-Based Research in Ghana, Uganda, and Guatemala

**DOI:** 10.5334/aogh.4951

**Published:** 2026-01-30

**Authors:** Efua Esaaba Mantey, Emilly Maractho, Erwin Calgua-Guerra, Daniel Doh, Guillermo Zea-Flores, Carolina López, Sirry Alang, Peter Donkor, Judith N. Lasker

**Affiliations:** 1 Department of Social Work, University of Ghana, Legon, Accra, Ghana; 2 Uganda Christian University, Mukono, Uganda; 3 School of Medicine, University of San Carlos of Guatemala, Guatemala City, Guatemala; 4 School of Allied Health, University of Western Australia, Perth, Australia; 5 Rafael Landívar University, Guatemala; 6 Independent researcher, Guatemala; 7 University of Pittsburgh, USA; 8 Kwame Nkrumah University of Science and Technology, Kumasi, Ghana; 9 Lehigh University, Bethlehem, PA, USA

**Keywords:** short-term medical missions, community needs, skills, decolonialization, sustainability

## Abstract

*Background:* Growing attention to the proliferation of short-term medical missions (STMMs) in the Global South has increasingly taken the form of critiques of inadequately prepared volunteers, lack of community control and continuity. In response, scholars and practitioners in high-income countries have created guidelines for best practices. These have rarely incorporated the views of host community members and leaders. While research has begun to address what host countries want from STMMs, these projects have also been carried out almost exclusively by scholars from the Global North.

*Objectives:* The aims were to provide additional insights into host views in three countries that are frequent destinations for STMMs and to explore the possibility that design and direction by host country researchers would yield new perspectives.

*Methods:* Scholars from Ghana, Uganda, and Guatemala designed and directed studies of medical staff, public officials, and patients from multiple locations around each country. Interviews and focus groups were carried out with a total of 129 people.

*Findings:* All three studies found widespread appreciation of STMMs for providing needed medical services. In Ghana and Guatemala, language differences were cited as a major barrier, while Ugandan participants criticized volunteers’ lack of skills and mismatch between their expertise and community needs. Ghanaian and Ugandan participants voiced resentment of patients’ preference for white volunteers and the arrogance of some visitors. The amount of time and effort required to host was a common theme.

*Conclusion:* The findings confirm the importance for STMMs of understanding the local health context and language and working collaboratively and respectfully with hosts. Direction by host country researchers enhanced the value in several ways, including access to officials and establishment of trust with interviewees.

## Introduction

Short-term medical missions (STMMs) have proliferated in recent decades and attracted growing attention in scholarly and popular literature. These programs, which send volunteers and students from higher to lower income countries for health-related activities, include a wide variety of sponsors, participants, and activities [1]. This attention has taken the form of admiration of volunteers but also a growing critique of unethical practices, undermining of host country professionals, and lack of effectiveness, suitability, sustainability, and cultural humility [2–9].

In response, scholars and practitioners have created guidelines recommending best practices for short-term programs [10]. However, critiques and guidelines are both produced almost entirely by people in high-income countries (HICs) and rarely incorporate the views of host community members and leaders [11].

A growing call for “decolonization” of global health emphasizes the importance of undoing the skewed power relationships between people from HICs and their hosts in LMICs [12–14]. These power relationships exist in decision-making about programs and also in research; in both cases, people in LMICs do much of the work while people in HICs retain control and acquire most of the credit and career advantages [15–20]. This paper addresses both elements of the decolonization critique programs and research by highlighting views of host country staff and patients regarding those programs and by having the design and direction of the research under the control of host country scholars.

The three studies reported here, in Ghana, Uganda, and Guatemala, are among the first, to our knowledge, to be designed, directed, and analyzed by host country researchers [21–23]. The goals of the three-country project were to obtain additional insights into host views and to explore whether studies carried out by scholars from the country yield different results.

## Background

In very recent years, a growing number of studies have considered host country views of these programs. This shift has obvious value, but as with the guidelines, almost none has been carried out by scholars in the host countries.

This bias is confirmed by two recent scoping reviews of peer-reviewed research published in English [24, 25]. The combination of results from Amick et al. and Lu et al. results in 33 unique articles that have a primary focus on the perceptions by host country individuals of short-term medical visits from HICs.

The 33 articles have a total of 176 authors and co-authors. One hundred forty-five (82.4%) of these authors are scholars who are based in HICs, although some are originally from LMIC countries. Only one article has a first author from the host country being studied and whose institutional home is also in that country [26]; 22 articles (two-thirds) have no authors at all from outside the HICs. None of the articles is published in a journal from outside the Global North. Almost all the studies were designed and received IRB approval in HICs.

The virtual absence of host country scholars in assessing STMMs calls for research based on the principles of community-based participatory research (CBPR). This now widely recognized approach requires that research questions, procedures, and analyses be driven by members of the community of interest [27, 28].

Control of research by the community is not only ethically responsible and more effective for planning; it is also likely to produce more valid results [29, 30]. When the project designers and often the interviewers are outsiders, risks to validity due to norms of politeness, legitimate concerns about possible threats to their positions, and the operation of the social desirability principle in interviewing [31] are intensified. There is logically little likelihood of a white foreigner accompanying, and occasionally even leading, a medical volunteer group, expecting hosts (many of whose livelihoods depended on these visits) to speak candidly about their concerns.

Rozier [32] confirms these concerns, finding that host community members may be reluctant to criticize STMMs in talking with researchers from other countries for fear of risking the income and jobs the programs bring. “Even more, in many cultures, being good hosts is a central value and so it would be anathema to critique a guest, even when their actions are harmful.”

Amick et al. also found evidence of reticence in critiquing STMMs: “many studies reporting local perceptions indicated that participants voiced concerns about appearing too critical of Global Health Engagements (GHEs) for fear of losing them.”

Alexander Weinrieb and colleagues [33, 34] have tested the once widely held norm that strangers can elicit more accurate responses than people known to interviewees. Their studies, as well as others [35, 36], confirmed in a variety of different LMICs the advantages of having interviewers who are socially close to study participants.

### Lessons from prior research on host views

Amick and colleagues’ recent review offers a useful summary of findings in the literature. They identified three analytical themes in coding the articles in their review, and we use that framework here to enumerate key findings in the literature to date.

The first theme, “resources and perceived benefits,” has two categories. “Tangible” resources and benefits include financial support, supplies, and human resources; “intangible” refers to enhanced reputations of individuals and institutions, relationship-building, and knowledge and skills.

The second theme, “perceived challenges,” is grouped into “attributes” and “actions” of the visitors. Undesirable attributes include the foreign volunteers’ arrogance and disrespect for host clinicians, inadequate skills, and cultural unpreparedness. “Undesirable actions” include engaging in practices that fall outside visitors’ skills and scope of practice, disregarding local practices, and exerting unwarranted power over local practitioners.

“Opportunities for improvement” included better planning, communication, and preparation of individuals before they travel. Host organizations should have increased say in determining needs and developing objectives, the selection and preparation of participants, and improved opportunities for monitoring, evaluation, and feedback after the visit. There was also a preference for participants to communicate in the local language.

In these and other studies, host organizations and staff express a desire for a far more significant role in short-term programs beyond providing logistical support and that their feedback be taken seriously by volunteer organizations [37]. Smaldino et al. [38] analyzed how perceived power differentials between host organizations and international visitors can make a true partnership difficult to achieve. These power differentials are reflective of much broader historical and current international inequalities.

The current paper has two goals: to add to the limited literature on host country attitudes towards STMMs by studying three countries that are frequent hosts to these programs and to consider how these findings might be different from those of previous studies led by foreign scholars. The project had an additional objective: to study country policies and regulations governing STMMs [39].

## Methods

Dr. Efua Mantey and Dr. Daniel Doh were P.I.s for the Ghana study, Dr. Emilly Maractho for Uganda, and Dr. Erwin Calgua Guerra and Dr. Guillermo Zea Flores for Guatemala. Mantey, Maractho, and Calgua recruited, trained, and supervised colleagues and graduate students to carry out the research. The P.I.s all brought to the project many years of research, teaching, and project supervision, including on health-related topics, in their own countries. Additionally, Dr. Calgua Guerra was involved as a host staff member in STMMs in Guatemala for many years.

In each country, the lead investigator(s) determined how to carry out the research, whom to interview and where, and analyzed and wrote up the results. The Ugandan and Guatemalan studies included patients, while in Ghana, patients were not included. Each of the studies was approved by a review committee in the relevant country (Ghana Health Service Research Ethics Board, Makerere University School of Social Sciences Research Ethics Committee, and ZUIGEME, a local Institutional Review Board in Guatemala) and also by the Institutional Review Board at Lehigh University, the funding source.

The Ghanaian researchers carried out in-depth interviews in 2018 with respondents purposively selected, after consultation with Ghanaian public health experts, from healthcare institutions in six regions across the country, to provide diversity in population size and healthcare availability.

The 24 interviewees (eight medical officers, seven nurses, seven administrators, and two regulators) worked in public, private, and faith-based facilities and for government entities. The lead author and two trained research assistants conducted the interviews in English, although the interviewers were familiar with the local language in each location [21].

### Uganda

The research team selected ten locations in Uganda to represent the country’s rural and urban regions. Graduate research assistants at Uganda Christian University were selected based on their knowledge of the specific regions chosen and the local languages in those areas, and they conducted and transcribed interviews with 46 people in 2019. These included 17 policymakers (Ministry of Health, District Health Services, Chief Administrators at Local Government, and Private Health Providers), 19 administrators and staff of host institutions (NGOs and media), and 10 host community members (Local Council representatives at the village level and patients who were participants in an STMM) [22].

### Guatemala

The P.I.s identified key informants from the Ministry of Public Health and Social Assistance (MSPAS) and the College of Surgeons of Guatemala (COLMED) in order to obtain a list of groups intending to visit Guatemala. Such programs are called JMCPs (Jornadas Medicas a Corto Plazo) or “jornadas.” From the list, two jornadas planned in rural areas and two in cities were randomly selected and contacted to request authorization to conduct the research. A program that had not registered with COLMED was also selected. Due to the COVID-19 pandemic, research could be conducted at only three of the planned locations. They were carried out during the conduct of STMMs in the months of January to March 2020.

A total of 40 individual interviews and six focus groups were conducted in three locations, all non-governmental hospitals founded by humanitarian and faith-based organizations. A total of eight interviews were conducted with healthcare staff and administrators and 32 with patients. The focus groups had a total of nineteen patients. Interviews and focus groups were carried out by two PhD-level Guatemalan social scientists. Patient interviews occurred in the waiting room during the intake process.

In cases where a Mayan language was identified, translators (provided by the communities) were included; most of the research was conducted in Spanish. Due to the COVID-19 pandemic, in one of the urban locations, it was only possible to conduct interviews, as focus groups were not allowed due to the possibility of transmission [23].

In all three countries, interviews and focus groups were tape recorded and transcribed, and each country team worked together to develop codes and analysis, using Nvivo 12.

Also, ethical issues of informed consent, confidentiality, voluntary participation, and anonymity guided the study. Participants were provided with information that identified the researcher, the purpose of the study, participants’ expectations, and how the research results would be disseminated to enable them to give their consent. Emphasis was placed on the fact that participation was voluntary, and participants could choose to withdraw at any stage of the interaction if they did not wish to continue.

In reporting the results of the study, the names of the participants were not disclosed in order to protect their identities and ensure their anonymity. Again, to ensure confidentiality, interviews were conducted at a place convenient for participants so that information shared by participants would not be heard by third parties. Also, derogatory names and demeaning remarks were not used to ensure that no harm was caused to participants involved in the study.

## Results

Due to differences in methodology, participant selection, and coding practices, the results cannot be directly compared to each other. However, there were several common themes. All of the studies cite the need for filling big gaps in health systems in many locations, due to shortages of personnel and equipment, substantial disease burden, and lack of adequate financial resources. Many respondents greatly appreciated the visiting health teams in light of these concerns.

### Advantages and challenges of STMMs

In all three countries, participants referred to improvements in health for patients. These were attributed to the role of what are perceived to be skilled practitioners, the use of modern equipment and supplies, the empathetic manner of the visitors, and greater access to care, financially and geographically. The government’s inability to provide services to all was an important theme.

Patients report that since they cannot afford the healthcare they need, or the medications prescribed by public facilities, their health condition deteriorates; it improves when volunteers bring free services, medicine, and equipment and make them available in rural and other underserved areas.

As a Ugandan high school teacher noted,

*Volunteers are generally highly energetic, they also bring with them new ideas and expert knowledge and offer services kindly at almost zero cost.*

Participants also had concerns about the programs and the volunteers. Language and communication problems dominate in the responses in Ghana and Guatemala, while the Ugandan participants were more concerned about the volunteers not having proper skills and motivations. Security and cultural issues emerged in comments from Ugandan participants, who were concerned about risks arising from the assumption that the visitors would have a lot of money, combined with their lack of attention to safety and their ignoring cultural norms around behavior and dress.

A concern that emerged from staff interviews was the amount of work and expense required to host visitors. One hospital administrator in Ghana said,

*We host them in a hotel, you must be prepared to feed them as well and this comes with a cost, but we see it as part of our Corporate Social Responsibility. Costs may include preparing for them and organising programmes.*

A Ghanaian nurse cited a different kind of work burden required to protect patients:

*These are people coming from other countries. Perhaps they may even fake their license, so that you cannot leave them alone to carry out responsibilities. Once I cannot leave the patient in your care to be solely responsible, then I do not see you to be too important. Work must go on with or without them.*

Additional costs cited were the need to help clear imported equipment through customs, only to find sometimes that it is not suitable or usable and has to be discarded.

Ugandan respondents were asked about what they themselves contribute to the operation of STMMs. Over half (52%) referenced support activities provided in advance of a visit, 28% mentioned the contribution of their experiences to the visitors, and 9% referred to the provision of welfare and security benefits. For example, one said,

*You know when a visitor visits your home you must be close to monitor progress or any challenge they are facing. In fact, when we receive these visitors we almost work 24 hours to make sure everything is going smoothly.*

Language was the primary challenge for Guatemalan respondents, impeding communication between volunteers and patients and their families. As one participant noted,

*The most difficult is sometimes the language. For example, there is an interpreter who speaks English, but there is no interpreter from Quiché to English. Communication to the volunteer, that is the most difficult part, and not only English to Spanish. In that case, there are two translators; we have to look for a person who can translate from the “vernacular” language into Spanish and then from Spanish to English. It’s hard, but you have to do it.*

A Guatemalan patient mentioned that he was given a box with tablets, but due to the lack of a translator, he did not understand what they were, what they contained, or how often they had to be ingested.

Some Ghanaian staff communicated their feelings of exploitation. For example, these participants felt that volunteers come to practice some skills that they might not be allowed to use in their home countries.

A Ugandan participant also felt unfairly treated:

*We have seen people who come, somebody is actually below you in terms of their academics, in terms of their skills, and the person comes and is structurally put above you.*

A number of Ghanaian staff members voiced frustration about patients’ preference for foreign practitioners, even when they are not as knowledgeable as the Ghanaian staff. This preference on the part of patients has several sources, including the free services and fresh energy the volunteers bring. But there is also a disturbing racial dimension:

*Because they see a white person, it will draw all of them there (Administrator).*

A Ugandan patient confirmed this perspective: “*I had a big wound that was not healing but when these volunteers, especially white people, came, I was treated. Now I’m working seriously on my farms and waiting for the season to start digging.*”

Another Ghanaian nurse explained that the preference is also due to the lighter workload for the visitors:

*One of the problems we face in Ghana is patient-to-nurse ratio; I was told that over there it is either 1 to 1 or 2 patients are assigned to 1 nurse. So just imagine the care that will be rendered here where you had about 20 patients assigned to 2 nurses. Regardless, we attend to the patients very well, and then a volunteer comes around and started showing him a little care. Because we do not give them much work to do when they come around, they seem to spend much time with the patients so the patients felt that they care so much for them.*

### Advantage of insider research

The studies revealed a number of valuable observations regarding how findings in a study carried out by in-country researchers differ from research designed and analyzed by outsiders. Guatemalan researchers noted that patients are used to being asked questions and even participating in research, so it is not too important who is conducting the project. On the other hand, some of the people interviewed were physicians and government officials, with education and status comparable to that of the researchers. An important advantage, however, noted by the Guatemalan team, has to do with the fact that authorities at the Ministry of Health and the College of Physicians knew the co-P.I.s (EC and GZL) personally over a long period of time; therefore, a trust was already established that helped in the interaction and in obtaining information about the jornadas. Further, because both P.I.s had many years of experience participating in STMMs, they were well aware of the different actors and norms related to STMMs in Guatemala.

No other studies, as far as we are aware, evoked as many comments about the burden of hosting. Even more unusual were the references to racism in this study. In Ghana, the investigators observed as follows: “The lack of respect for the expertise of host country practitioners, arising from the ignorance and biases of visiting volunteers as well as the colonialized attitudes of patients, has been cited elsewhere but is more prominently mentioned here…” [23].

Ghanaian participants often emphasized the value of their own experience and superior ability to provide care with limited means and for conditions they know better. Notable are instances reported of convincing the visitors of their capabilities. Thus, while the issue of lack of respect that is often cited in other studies is apparent here as well, there were a number of expressions of pride at changing those attitudes, a valuable finding not noted elsewhere.

For example, a Ghanaian surgeon said: *Do they feel that they know too much or they are coming with exceptional skills? Maybe when they are coming they will feel so but after sharing knowledge, then they realize that we know what we are about.*

Perhaps the most striking example comes from a hospital administrator:

*Everyone wants to go to Africa to see the black monkeys here [Laughing...] In fact, they come here to see that we are no monkeys after all. You understand. When they come, they marvel at the level of expertise of the Ghanaian doctors and nurses. Sometimes our guys teach them some of the things that needed to be done* [23].

A similar sentiment was voiced by a Ugandan physician:

*If you’ve got people who have not yet got much exposure relating with people, everything which is “un-European,” “un-American” looks funny. Like questioning your culture, your way of life, and people seem to appear like they are animals, that whatever you are doing, you are not providing services but you are especially coming to show how better you are to them.*

Ghanaian interviewers noted that participants often switched to the local language to answer questions asked in English, even though the latter is Ghana’s official language and all participants were fluent in English. This tendency, along with side comments such as *you understand*, as seen in the quote above, or *You know these white people, *or referring to the interviewer as *my sister* indicates a level of openness and comfort that is necessary for research. Ghanaian researchers reported that participants were comfortable speaking with them because they shared a perspective (“you understand”) and often a local language.

## Discussion

The study examined host country perspectives on STMMs in three countries. Specifically, it explored how local researchers and other stakeholders perceive and understand ethical considerations and their long-term influence on community members and the health systems in these three countries.

An important limitation of almost all the work on this subject, including the studies reported here, is the short-term nature of the researchers’ involvement with participants. Almost all of the prior studies relied on one-time in-person interviews or written or online questionnaires. Additionally, we have to consider the possible influence on the study of its being funded by a North American university and based to some extent on a previous study by a North American researcher (Lasker [1]). Yet in each case, the P.I.s decided independently on the methodology for recruiting respondents, on the questions to be asked, and on the analysis undertaken. They also selected and trained interviewers.

The “insider” versus “outsider” researcher is, of course, not a simple dichotomy. Each of these three studies was designed and led by a doctoral-level faculty member based at a university in or near the country’s capital, and the interviewers were all master’s or doctoral-level students/researchers at those universities. The use of the insider helped establish an easy rapport with participants and deal with all ethical concern issues. Acceptance was high and trust was developed quickly, which allowed participants to open up to the researchers and express their opinions freely. For example, participants comment like “*Everyone wants to go to Africa to see the black monkeys here [Laughing...] In fact, they come here to see that we are no monkeys after all*.”

This comment is a very powerful evocation of the many positive as well as troubling dimensions of STMMs. It is also the most obvious example of the advantage of the study being carried out by Ghanaians. It is unlikely that such a comment would have been expressed in this manner to a non-African interviewer. Indeed, a similar reference to white people considering Ghanaians to be “monkeys” appeared in an earlier interview carried out in Ghana by a Ghanaian-American (Lasker [1]); we have seen nothing comparable in other research reports. This boldness from the participants indicates confidence in the researcher, which will lead to sharing sensitive information without fear of judgment since they believed we share their background and understand the issues. Also, the participants felt safe around us since they believed we were familiar with the cultural settings and the issues being discussed.

The CBPR model, to be followed more fully, would have meant active involvement of relevant staff (and patients if part of the study) in the design, execution, and analysis of the research.

It is also important to acknowledge the great variety within STMMs, limiting the generalizability of findings. Brief visits from higher to lower income countries can range widely in type, purpose, and location. On one end, there are the one-time arrivals of groups of mostly untrained individuals who set up different kinds of services with no local partner, needs assessment, continuity, or evaluation. On the other end are long-standing partnerships between medical institutions in which host staff define needs and are engaged in mutual learning. Often these have year-round programs in which short-term visits by students or by medical specialists are just one element. Future research is needed to identify differing responses by local communities to these different kinds of programs.

There were limitations specific to the Guatemala study. While carrying out the interviews at sites in Guatemala, researchers noticed that participants’ answers were usually short and direct. The interviews took place during the period of time when patients were waiting to be examined by the medical team to know if they were suitable for surgery or medical care. (Due to the Guatemalan context in regard to insecurity, participants usually do not feel comfortable being approached in their homes.) It is possible that the information obtained was limited due to the short available time in that precise moment, and perceptions that participants could have in regard to sharing something negative about the jornadas, which could hinder the possibility of being cared for. In addition, some of the interviews took place during the time when the COVID-19 pandemic was beginning, with the attendant fears of contagion.

Many of the findings from the three studies reported here are consistent with those of previous studies. On the positive side, patients especially are very happy with the possibility of receiving medical care that might otherwise not be available or affordable, and staff appreciate the opportunity to provide services, to learn, and to enhance their own program’s status and capacity.

With regard to disadvantages, patients mostly want to be able to communicate with the visitors in their own languages. Quality of care is known to be diminished by poor communication between patients and practitioners [40–43].

Staff had many more concerns, although language was also the number one issue for staff interviewed in Ghana. While they acknowledge the potential benefits, they also cite the extra work they incur hosting and training outsiders, who mostly have little understanding of the medical and cultural context and yet may consider themselves (and be considered by patients) to be superior.

While recognizing the social, and in some cases, the linguistic gap between researchers and participants, we consider these studies to be a valuable but still preliminary step towards a more “insider” approach, one that is suggestive of its advantages over much previous research. Further research on this topic might compare results from interviewers with differing backgrounds and familiarity with communities under study.

## Conclusions

The three reports come to somewhat similar conclusions, although with different emphases resulting from differences in the countries, in participant composition, and in methodologies. It is notable that the country with the greatest percentage of patients in the study, Guatemala, also had the highest proportion of positive comments. Only in Ghana, where there were no patients involved, did the number of challenges mentioned outnumber the mentions of advantages.

Studies of patient satisfaction with STMMs are rare and require considerably more attention. Patients who seek care from an STMM generally do so because they cannot afford or do not have access to better alternatives; it is understandable that they would be appreciative. We also recognize that interviews and focus groups carried out with patients at the site of a clinic are likely to yield more positive responses. The comments about kindness and patience from Guatemalan patients reflect previous findings [7] that short-term volunteers generally bring new energy and interest to a situation in which host staff have been working for a long time and often in difficult circumstances. Even with these positive views, we note a big concern about the language gap between volunteers and patients.

Medical staff, in contrast, experience more of the challenges of STMMs, such as the considerable work of hosting, the arrogant attitudes and assumptions about their inferiority made by some outsiders (who often know less), and misbehavior or lack of interest on the part of some volunteers. There is also some resentment when patients prefer the outsiders. As one Ghanaian nurse noted,

*There is a saying that “a prophet is never accepted in his own town.” You could not appreciate your fellow countryman who took care of you throughout your stay, but you extremely appreciate a volunteer who came to meet you for just a few days.*

The results of this study confirm recommendations made in prior critiques and in guidelines [10] that visiting volunteers and students should have the requisite skills, knowledge, and experience to meet the needs of the identified country and community, as determined by those communities. A hospital administrator in Uganda emphasized the importance of this point:

*The person who suffers the pain, is the one who knows the magnitude of the pain so I wouldn’t think a volunteer from outside would understand my problem more than me. Maybe you don’t have the money, the means, or you’re not the key decision person, but you know the problem. That is why most of the successful programs are those initiated, owned or originated by the local people.*

Many actions can be taken to enhance the value of STMMs. This can be achieved by improving communication at the practice level, fostering intercultural communication, and setting up expectations on both sides. Instituting long-term partnership mechanisms for collaboration between countries of origin and host countries at an institutional level and building the capacity of local experts through mentorship, research, and training can greatly enhance the value of STMMs that could then be more effectively embedded in such longer term programs. The three studies [39] also found that stricter enforcement of host country licensing laws is needed. STMM participants from HICs should ideally have some language facility and also must show respect for their hosts’ capabilities and experience.

Sharma and Sam-Agudu [14] aptly point out that the work of decolonizing unequal power relations must be done largely in the Global South. This call for a shift, seen as well in a number of interviews with staff, would be enhanced by host countries taking control of medical activities that involve visitors from outside in order to align them with national goals and standards for the benefit of their populations. Visitors from higher income countries, as power holders by virtue of resources, have a responsibility to seek out and follow host country regulations and standards. Frequent communication and guidance from host country’s officials in the context of a mutually respectful partnership is necessary to enhance the quality and impact of STMMs.

It is clear that the country hosts take pride in their own abilities to provide quality care, and that they want to be recognized for these abilities and for what they teach the visitors. As described by a Ugandan physician,

*It has to be a win-win sort of thing that we will all be at an equal level; first we should recognize that we are benefiting from each other… these whites benefit by coming to us and also by learning a lot from us and so we can enhance that when they come and we share.*

## Data Availability

We do not wish for the dataset to be accessible to a wider audience; however, requests for the dataset via email (efuam@yahoo.com) will be considered and materials provided on a case-by-case basis.
